# Susceptibility of Asialoglycoprotein Receptor-Deficient Mice to LPS/Galactosamine Liver Injury and Protection by Betaine Administration

**DOI:** 10.3390/biology10010019

**Published:** 2020-12-31

**Authors:** Karuna Rasineni, Serene M. L. Lee, Benita L. McVicker, Natalia A. Osna, Carol A. Casey, Kusum K. Kharbanda

**Affiliations:** 1 Research Service, Veterans’ Affairs Nebraska-Western Iowa Health Care System, Omaha, NE 68105, USA; karuna.rasineni@unmc.edu (K.R.); bmcvicker@unmc.edu (B.L.M.); nosna@unmc.edu (N.A.O.); ccasey@unmc.edu (C.A.C.); 2 Department of Internal Medicine, University of Nebraska Medical Center, Omaha, NE 68198, USA; Serene.Lee@med.uni-muenchen.de; 3 Department of Biochemistry and Molecular Biology, University of Nebraska Medical Center, Omaha, NE 68198, USA

**Keywords:** lipopolysaccharide, galactosamine, apoptosis, betaine, fulminant liver failure

## Abstract

**Simple Summary:**

Previous studies from our laboratory have shown that chronic ethanol exposure-induced increase in apoptotic hepatocellular death is closely related to the ethanol-induced impairment in asialoglycoprotein receptor, a major component of liver sugar recognition system. The aim of this study was to examine whether the absence of this receptor confers increased susceptibility to fulminant liver failure induced by lipopolysaccharide/galactosamine. We further investigated whether treatment with betaine, a naturally occurring tertiary amine, prior to lipopolysaccharide/galactosamine injection is protective. Lipopolysaccharide/galactosamine injection caused a more pronounced liver damage in asialoglycoprotein receptor-deficient compared with the wild-type control mice. In addition, prior administration of betaine was found to significantly attenuate the lipopolysaccharide/galactosamine-induced increases in several liver injury parameters. Our work underscores the importance of normal functioning of asialoglycoprotein receptor in preventing severe liver damage and signifies a therapeutic role of betaine in prevention of liver injuries from toxin-induced fulminant liver failure.

**Abstract:**

Background: Work from our laboratory has shown that the ethanol-induced increase in apoptotic hepatocellular death is closely related to the impairment in the ability of the asialoglycoprotein receptor (ASGP-R) to remove neighboring apoptotic cells. In this study, we assessed the role of ASGP-R in fulminant liver failure and investigated whether prior treatment with betaine (a naturally occurring tertiary amine) is protective. Methods: Lipopolysaccharide (LPS; 50 μg/kg BW) and galactosamine (GalN; 350 mg/kg BW) were injected together to wild-type and ASGP-R-deficient mice that were treated for two weeks prior with or without 2% betaine in drinking water. The mice were sacrificed 1.5, 3, or 4.5 h post-injection, and tissue samples were collected. Results: LPS/GalN injection generate distinct molecular processes, which includes increased production of tumor necrosis factor-α (TNF-α) and interleukin-6 (IL-6), thus causing apoptosis as evident by increased caspase-3 activity. ASGP-R deficient animals showed increased liver caspase activities, serum TNF-α and IL-6 levels, as well as more pronounced liver damage compared with the wild-type control animals after intraperitoneal injection of LPS/GalN. In addition, prior administration of betaine was found to significantly attenuate the LPS/GalN-induced increases in liver injury parameters. Conclusion: Our work underscores the importance of normal functioning of ASGP-R in preventing severe liver damage and signifies a therapeutic role of betaine in prevention of liver injuries from toxin-induced fulminant liver failure.

## 1. Introduction

Fulminant liver failure results from massive hepatocyte death and severely impairs liver functions. This clinical syndrome has a high mortality rate despite several options available such as liver support systems and liver transplantation [1,2,3,4].

A well-established and widely used experimental model of fulminant hepatic failure is the combined administration of lipopolysaccharide (LPS) and galactosamine (GalN) [5,6,7,8]. Both inflammation and apoptosis are consistently observed in a number of inbred and outbred strains tested with this model [9,10]. Further, distinct molecular processes have been identified in LPS/GalN liver injury. It has been shown that the metabolism of GalN leads to hepatotoxicity through inhibition of mRNA and protein synthesis; this occurs due to the concurrent accumulation of UDP-GalN derivatives and a depletion of hepatic UTP [11]. This GalN “priming” leads to the potentiation of the toxic effects of LPS to produce typical hepatic cell injury followed by fulminant liver failure within 4–6 h of LPS/GalN administration [12,13]. In particular, TNF-α production by the LPS-activated Kupffer cells (KC) that primarily causes apoptosis of GalN-“primed” hepatocytes at an early stage in LPS/GalN-induced liver injury [14,15]. TNF-α is also responsible for the upregulation of the adhesion molecules, CXC chemokine formation, the activation of polymorphonuclear neutrophils (PMNs), and their sinusoidal accumulation [16]. GalN/TNF-α also induces gap formation in endothelial cells, which allows PMNs to recognize apoptotic hepatocytes. This is the trigger for PMN extravasation and attack on injured cells. These events aggravate and accelerate parenchymal cell injury leading to necrosis of hepatocytes at a later stage [17,18]. In addition, the failure of the sinusoidal endothelial cells (SEC) barrier causes hemorrhage in the liver resulting in hypovolemic shock, which eventually kills the animal [19].

It has been shown that even if CXC chemokine formation and the PMN attack is prevented, the apoptosis continues to increase and eventually similar extent of injury is observed [16,20], signifying apoptosis as being germane to LPS/GalN-induced fulminant liver failure.

Our previous research has shown that the function of the asialoglycoprotein receptor (ASGP-R), a major component of liver sugar recognition system, is impaired following chronic exposure to ethanol. Further work in our laboratory has shown that the increase in hepatocellular apoptosis observed after ethanol consumption is closely related to the impaired ability of the ASGP-R to remove apoptotic cells; one of the most important physiological ligands of this receptor [21,22]. The generation of the ASGP-R deficient mouse strain has provided us with a tool to understand the physiological role of ASGP-R. Since hepatocellular apoptosis has been shown to be a major player in LPS/GalN injury, one of the aims of this study is to assess if the loss of ASGP-R makes these mice more susceptible than wild-type controls to LPS/GalN-induced fulminant liver failure.

Only 14% of patients diagnosed with fulminant liver failure recover with medical therapy. While orthotopic liver transplantation has improved the chances of survival of patients with fulminant liver failure, there is a high risk associated with immunosuppressive agents used in transplant patients [1,2]. Therefore, there is a need to identify anti-apoptotic agents that could be effectively used to treat fulminant liver failure. Several studies conducted in our and other laboratories have shown that betaine can mitigate liver diseases of diverse etiology such as alcohol [23,24,25,26,27,28,29,30,31,32,33,34], carbon tetrachloride exposure [35,36,37,38], high-caloric intake, and metabolic syndrome related [39,40,41,42,43,44,45]. There are also numerous publications demonstrating betaine’s protective role in apoptosis [24,46,47,48]. Thus, we also wanted to examine whether prior administration of betaine could protect these ASGP-R-deficient mice from developing severe liver injury following LPS/GalN injection.

## 2. Materials and Methods

### 2.1. Animals

Wild-type C57Bl6/129SV F2 cross mice (WT) and ASGP-R-deficient (RD) mice of the same strain were obtained from Jackson Laboratories (Bar Harbor, ME, USA). As described in our earlier publications, RD mouse was developed by abolishing MHL-2 gene that encodes the minor subunit of ASGP receptor [49,50]. RD mice do not show any phenotypic abnormalities. Female mice (20–22 g) were fed with 2% betaine in drinking water or water alone for 2 weeks and then randomized to be injected intraperitoneally with either saline or a solution of LPS (E.coli 026:B6, Sigma# L2654; 50 μg/kg BW) and GalN (350 mg/Kg BW). The mice were sacrificed 1.5, 3, or 4.5 h post-injection [5,6]. At necroscopy, the liver was removed, and portions were either immediately processed for histological examination or snap frozen in liquid nitrogen and stored at −70 °C for caspase enzyme analysis. Blood samples were collected via the axillary artery and the prepared sera were stored at −70 °C until analyzed for AST/ALT activities and cytokine levels. The care and use of these mice and the procedures performed on them were approved by the Institutional Animal Care and Use Committee at the Omaha Veterans Affairs Medical Center.

### 2.2. Serum Transaminase

Serum alanine aminotransferase (ALT)/aspartate aminotransferase (AST) levels were determined using the VITROS 5.1 FS Chemistry System (Ortho Clinical Diagnostics, Raritan, NJ, USA).

### 2.3. Serum Cytokine Levels

Tumor necrosis factor-α (TNF-α) and interleukin-6 (IL-6) levels were measured using the BD OptEIA Mouse TNF-α and IL-6 ELISA kit purchased from BD Biosciences, San Diego, CA, USA.

### 2.4. Histopatholog

Paraffin-embedded liver tissue sections were processed for hematoxylin/eosin staining for histological evaluation. The severity of liver injury was assessed by evaluating portal inflammation, hepatocellular necrosis, inflammatory cell infiltration, and loss of cell architecture.

### 2.5. TUNEL Assay

The formalin-fixed liver tissue was also processed and stained with terminal deoxynucleotidyl transferase-mediated dUTP nick-end labeling (TUNEL). The number of apoptotic hepatocytes was quantified based on positive TUNEL staining and morphological criteria. Apoptotic hepatocytes were counted individually for five independent sections and expressed as the average number of TUNEL-positive cells per microscopic field.

### 2.6. Caspase-3 Activation Assay

Portions of frozen livers were processed and then assayed for specific caspase activities following manufacturers’ instructions using commercially available caspase-3 fluorogenic substrate, Ac-DEVD-AMC (BD Biosciences, San Diego, CA, USA). Caspase activity was evaluated by measuring the release of AMC (7-Amino-4-methylcoumarin) obtained by the cleavage of the defined synthetic peptide sequence by caspase-3, using a Perkin Elmer Luminescence Spectrophotometer LS 50B. Free AMC obtained from Sigma-Aldrich (St Louis, MO, USA) was used as the standard. Protein was determined using the BCA Protein Assay kit from Pierce. The caspase-3 activity was expressed as pmoles of AMC produced per mg liver protein.

### 2.7. Statistical Analyses

The results were presented as mean ± SEM. Data were analyzed by one-way ANOVA, followed by Tukey test. A *p* value < 0.05 was regarded as statistically significant.

## 3. Results

### 3.1. Histological Changes

**Figure 1:**
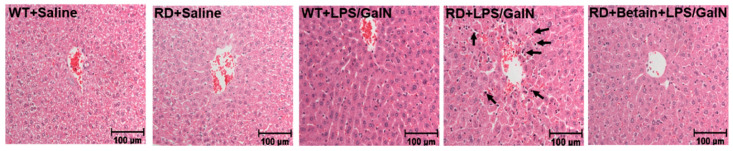
Histology of livers from wild-type (WT) and asialoglycoprotein (ASGP) receptor-deficient (RD) mice. Paraffin embedded sections were prepared and stained with hematoxylin and eosin stain (H & E). Photographs (200× magnification) show representative liver sections of saline or lipopolysaccharide (LPS)/galactosamine (GalN)-injected WT and RD mice. After 4.5 h of LPS/GalN injection, RD mice showed increased apoptotic hepatocytes (arrows) and areas of hemorrhage, which were reduced with betaine pretreatment.

Histological analysis of WT mice injected with saline or sub-lethal dose of LPS/GalN revealed a normal liver lobular architecture. (Figure 1). The same features were also seen in RD mice injected with saline. However, the mice injected with LPS/GalN showed areas of portal inflammation and apoptotic hepatocytes, which was randomly distributed throughout the parenchyma. These mice also had a moderate increase in inflammatory cell infiltration and hemorrhage. These pathological changes were ameliorated by betaine pretreatment.

### 3.2. Serum ALT and AST Levels

**Figure 2:**
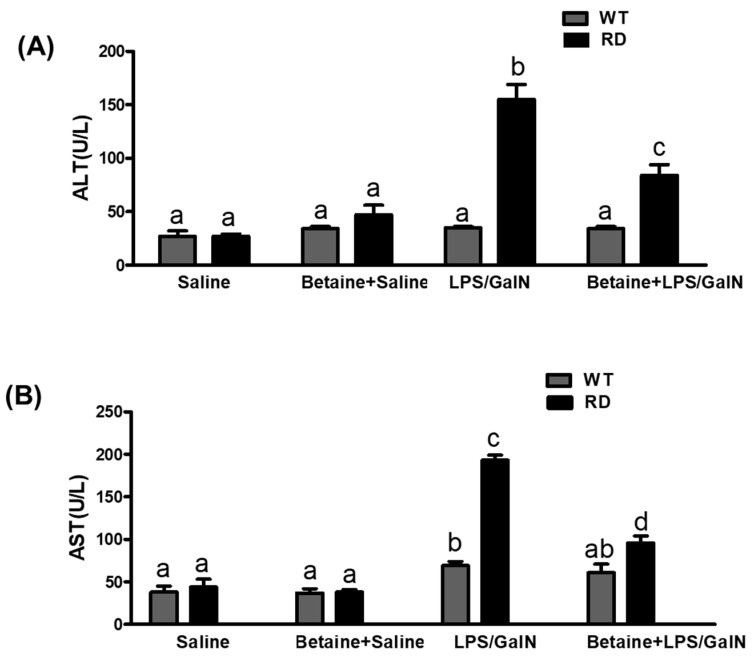
Effect of LPS/GalN and betaine on (**A**) serum alanine aminotransferase (ALT) and (**B**) aspartate aminotransferase (AST) levels. Values are means ± SEM (n = 8). Values not sharing a common letter are statistically different, *p* < 0.05.

ALT and AST levels, serum markers of hepatocyte injury, were within normal range in the saline-treated WT and RD mice at all time-points examined. Both WT and RD animals injected with LPS/GalN showed increases in serum liver injury markers only at 4.5 h post-injection. Whereas the WT exhibited a modest (< than 2-fold) increase in AST levels only, RD mice showed an over4-fold increase in both AST and ALT levels compared to saline-injected miceBetaine pretreatment significantly attenuated the AST and ALT levels in LPS/GalN-injected RD mice (Figure 2).

### 3.3. Cytokine Levels

**Figure 3:**
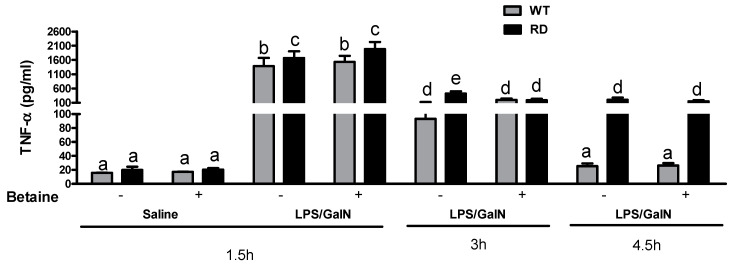
Effect of LPS/GalN and betaine on serum TNF-α levels. ELISA analysis of TNF-α concentration changes in serum at 1.5, 3, and 4.5 h after saline or LPS/GalN injection. Values are means ± SEM (n = 8). Values not sharing a common letter are statistically different, *p* < 0.05.

We observed TNF-α serum levels peaked at 1.5 h post-LPS/GalN injection and RD mice showed significantly higher TNF-α compared to the WT mice under the same conditions. These levels declined at 3 h in both WT and RD mice and reached to baseline at 4.5 h post LPS/GalN injection in WT animals but not in RD animals. Betaine treatment did not attenuate the LPS/GalN induced increase in TNF-α levels in either the RD or WT mice (Figure 3).

**Figure 4:**
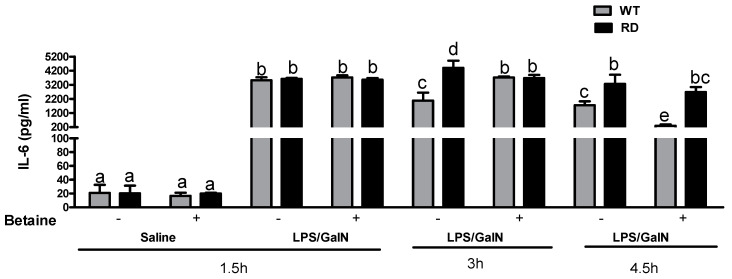
Effect of LPS/GalN and betaine on serum IL-6 levels in WT and RD mice. ELISA analysis of IL-6 concentration in serum at 1.5, 3, and 4.5 h after saline or LPS/GalN administration in WT and RD mice with or without betaine pretreatment were measured. Values are means ± SEM (n = 8). Values not sharing a common letter are statistically different, *p* < 0.05.

Contrary to the serum TNF-α profile, the increases in serum IL-6 levels were sustained for a longer period after LPS/GalN injection in both the WT and RD mice. However, the levels of IL-6 were higher in RD mice at 3 and 4.5 h after LPS/GalN injection relative to the levels observed in WT mice under the same conditions. Betaine partially attenuated the IL-6 release in RD mice at these later time points (Figure 4).

### 3.4. Hepatocyte Apoptosis

**Figure 5:**
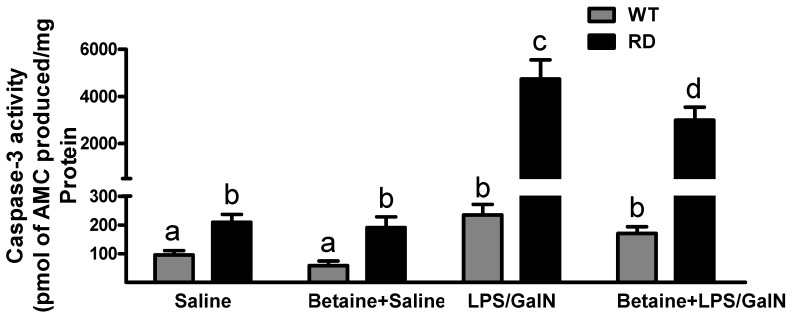
Caspase-3 activation in WT and RD mice at 4.5 h post injection. WT and RD mice treated with or without betaine treatment were injected with saline or LPS/GalN. Hepatic caspase-3 activity was evaluated by measuring the release of 7-amino-4-methylcoumarin (AMC) obtained by the cleavage of the synthetic peptide sequence by caspase-3 in liver homogenate. Values are means ± SEM (n = 8). Values not sharing a common letter are statistically different, *p* < 0.05.

Caspase-3 activity was at baseline at all times in the liver of saline-injected WT and RD mice. A significant increase in caspase 3 activity was noted in RD mice only at 4.5 h after LPS/GalN injection. In contrast, only a modest increase in caspase-3 activity in WT mice injected with LPS/GalN at 4.5 h post-injection. This increase was attenuated in betaine pretreated RD mice (Figure 5).

**Figure 6:**
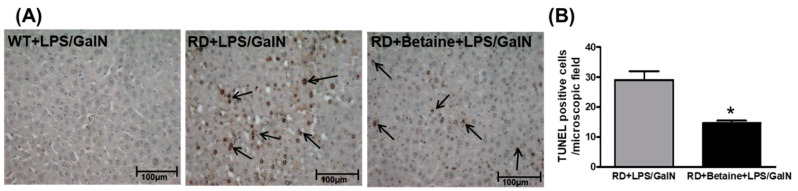
Apoptosis in livers of WT and in RD mice after 4.5 h post-LPS/GalN injection. Apoptosis was detected by terminal deoxynucleotidyl transferase dUTP nick end-labeling (TUNEL) in liver section. (**A**) Photographs (200× magnification) show representative liver sections of WT, RD, and betaine-treated RD mice at 4.5 h of LPS/GalN injection. TUNEL stain (arrows) showed increased liver cell apoptosis in the representative RD mouse after 4.5 h of LPS/GalN injection, which was significantly reduced in the betaine-pretreated LPS-GalN-injected RDI mouse. (**B**) Number of TUNEL positive cells/microscopic field. Results are from 10 fields/tissue section, and data pooled from 4 animals/group. Values are mean ± SEM; * *p* < 0.05.

Apoptotic nuclei were also detected by TUNEL staining. No TUNEL staining was seen in liver slices of WT mice injected with saline or LPS/GalN at 1.5, 3, or 4.5 h post-injection. Further, no staining was observed in RD mice injected with saline at all time points studied or following LPS/GalN injection at 1.5 or 3 h post-injection. However, many TUNEL positive hepatocytes were observed in RD mouse liver tissue 4.5 h post-injection with LPS/GalN. A few positive cells were seen in livers form the mice pretreated with betaine (Figure 6).

## 4. Discussion

The cascades of events that occur following LPS-GalN administration are well documented. This model of liver injury takes advantage of the ability of the GalN to inhibit transcription and thus to potentiate the toxic effects of LPS to produce typical hepatic cell injury followed by fulminant liver failure within 4–6 h of LPS/GalN administration [51,52]. In particular, hepatocellular apoptosis has been shown to be an integral part of LPS/GalN-induced liver injury [53]. In this study, we utilized LPS/GalN model of liver injury to study the physiological role of ASGP-R. Several features of ASGP-R function prognosticate its therapeutic role in the context of chronic liver diseases. Most important is its role in the phagocytosis of apoptotic bodies [21,22], which by precluding their uptake by the non-parenchymal cells, prevents activation of Kupffer/hepatic stellate cells and liver damage progression [54,55].

In this study, we used a sub-lethal dose of LPS/GalN, which was half of what has been used routinely for inducing fulminant liver failure. While this dose of LPS/GalN induced a TNF-α peak 1.5 h post injection in WT mice, we observed modest liver injury in this strain of mice by numerous criteria employed in this study. In contrast, the RD mice were susceptible to this sub-lethal dose of LPS/GalN and exhibited considerable liver injury only at 4.5 h post-injection. This was evidenced by several parameters such as elevated levels of plasma AST and ALT levels (Figure 2), increased liver caspase-3 activity (Figure 5), and considerable cell injury at histological examination by TUNEL-positive test (Figure 6). Further, there appeared to be a temporal sequence of events with TNF-α being the trigger (peak circulating levels of TNF-α was observed 1.5 h post-injection) to induce apoptosis of GalN-primed hepatocytes and the ensuing liver damage in RD mice. Recognizing that apoptosis is the major player in LPS/GalN-induced liver injury [53] and that ASGP-R plays an important role in removing apoptotic bodies [21,22], these results underscore the physiological importance of ASGP-R in maintaining homeostasis in the liver to prevent the cascade of events that generate liver damage. The dose of LPS/GalN used in this study produced only modest liver injury in WT mice despite a robust peak observed of TNF-α and IL-6 at 1.5 h post-injection of LPS/GalN. This is because the normally functioning ASGP-R prevented the buildup of apoptotic bodies and thereby also prevented the initiation of the cascade of events to produce fulminant liver failure. However, in RD mice, the absence of functional ASGP-R makes these mice susceptible to the sub-lethal dose of LPS-GalN.

Our laboratory has been studying the protective effects of betaine in a variety of animal and cell-culture models [24,25,26,28,29,30,31,32]. Betaine can prevent/protect against apoptosis induced by an array of agents [24,46,47,48]. Strategies increasing the anti-apoptotic armamentarium of hepatocytes have been shown to beneficially impact the outcome of fulminant hepatic failure [56,57,58,59,60,61,62]. In this study, we show that betaine is also effective in protecting against LPS-GalN-induced apoptosis in RD mice. While significant attenuation in caspase-3 activation, AST, and ALT levels were observed in RD mice pretreated with betaine, we observed no change in toxin-induced increases in serum TNF-α level. This indicated that the protective effects of betaine are downstream of TNF-α action. Similar results have also been reported by other investigators who showed the while glycrrhizin or A20 expression protected the livers from LPS/GalN induced liver damage, this occurs in the absence of significant differences in cytokine levels including TNF-α [56,63]. While the deleterious effects of TNF-α as a proximal mediator of hepatotoxicity are demonstrated in several models of liver injury including LPS/GalN [8,53], it also a co-mitogen required for proliferation of hepatocytes during liver regeneration, which emphasizes its beneficial effects. Thus, it is possible that maintaining high levels of TNF-α while protecting hepatocytes from apoptosis must be beneficial for liver regeneration and recovery.

To explore the mechanism of betaine action, we looked at the levels of metabolites of the methionine metabolic pathway in the livers of the WT and RD mice with or without betaine treatment and found no difference in the ratios of two important metabolites S-adenosylmethione (SAM) and S-adenosylhomocystiene (SAH), which is reflective of the methylation potential [25,29,31]. However, it appeared that the SAM levels were depleted faster in the RD mice as compared to RD mice that were pretreated with betaine (data not shown). SAM is the primary methyl donor in anabolic metabolism, serves as a precursor for glutathione (GSH), and is synthesized by the enzyme methionine adenosyltransferase (MAT) [64]. It has been reported that GalN treatment produces a substantial decrease in cellular SAM levels in hepatocytes by inhibiting MAT activity [65,66]. We have previously shown that betaine feeding can dramatically increase SAM levels in livers of rat [25]. We believe that betaine pretreatment prevents SAM depletion and thereby prevents the hypomethylation of ribosomal RNA induced by GalN treatment and the “priming” of hepatocytes to subsequent damage [67,68]. While many agents have been used to ameliorate LPS/GalN liver damage [56,57,58,59,60,61,62,63,69,70,71,72], betaine is inexpensive, bioavailable [73,74], and has no consistent relation with cancer, cardiovascular risk, or risk factors [75], making it a safe therapeutic.

## 5. Conclusions

To summarize, this study underscores the importance of normal ASGP-R function in liver homeostasis. By removing the apoptotic bodies effectively, ASGP-R thereby prevents the cascade of events leading to severe liver injury. Further in the event of impaired ASGP-R function, the administration of betaine can prevent the progression of the severe liver injury by preventing the activation of caspase-3.
